# Functional Outcome of Complex Elbow Fracture Managed With the Boyd Approach

**DOI:** 10.7759/cureus.52993

**Published:** 2024-01-26

**Authors:** Arnab Sain, Sitender Garg, Kanishka Wattage, Ahmed Elkilany, Arsany Metry, Nauman Manzoor

**Affiliations:** 1 Orthopaedics, All India Institute of Medical Sciences, New Delhi, IND; 2 Orthopaedics, Worthing Hospital, University Hospitals Sussex National Health Service (NHS) Trust, Worthing, GBR; 3 Orthopaedics and Trauma, Worthing Hospital, University Hospitals Sussex National Health Service (NHS) Trust, Worthing, GBR; 4 Orthopaedics and Trauma, Leeds Teaching Hospitals National Health Service (NHS) Trust, Leeds, GBR

**Keywords:** internal fixation, open reduction, functional outcome, elbow fracture, boyd approach

## Abstract

Introduction: The Boyd approach allows excellent access to the elbow and is used to treat complex elbow injuries using a single incision approach.

Materials and methods: In this study, we retrospectively evaluated 16 patients with complex elbow injuries treated with open reduction and internal fixation using the Boyd approach between 2016 and 2018.

Results: All fractures were well united in anatomical position. Postoperatively, the range of motion was not significantly different between the affected and unaffected elbows. The mean Mayo Elbow Performance Index score was 95 ± 5 (range 90 to 100). All study participants had satisfactory results and recovered to full activity. There was no incidence of posttraumatic arthritis of the elbow joint or synostosis of the radius and ulna.

Conclusion: Thus, according to our study, the Boyd elbow approach is a safe and effective method of treating elbow injuries.

## Introduction

There are numerous approaches to the elbow joint that are described in the orthopedic literature. Multiple approaches have been used in the past to expose the ulnohumeral, radiocapitellar, and proximal radioulnar joints to complex traumatic elbow injuries. Kaplan's approach is a lateral approach, and Kocher's approach is a postero-lateral approach for the management of elbow injuries. In 1940, the Boyd approach was described as a posterior approach, and it had the advantage of allowing good exposure of the elbow and was very useful for treating complex elbow injuries using a single incision approach [1-5]. It has the advantage of reducing operative time and also avoiding the need for repositioning, as it allows universal access to the elbow, which is useful, particularly in polytrauma or frail elderly patients. This approach also preserves all the important structures, nerves, and blood vessels around the elbow when using the proper technique. The versatile nature of the Boyd approach makes it a highly preferred approach among elbow surgeons, especially when dealing with complex elbow injuries. During the Boyd approach, the posterior interosseous nerve (PIN) needs to be protected as it is located near the site of exposure [1-5]. This study is aimed at checking the functional outcome in patients who had complex elbow injuries and underwent open reduction and internal fixation using the Boyd approach.

## Materials and methods

In our study, we retrospectively evaluated 16 patients with a combined olecranon fracture and a radial head or neck fracture treated with open reduction and internal fixation using the Boyd approach in 2016-2018 at the All India Institute of Medical Sciences, New Delhi, which is a tertiary care center in India. The patient selection was done randomly based on the period during which they had treatment in the hospital, and selection bias was minimized. The patients were aged 18-70 years. Preoperative consent was obtained from all patients, and ethical approval was obtained from the Ethics Committee of All India Institute of Medical Sciences (approval number: AIIMSA00808). There were six patients with a fall from a height and 10 patients with a motor vehicle collision. We excluded patients with head injuries.

In all the patients, preoperative and postoperative clinical and radiological examinations were done. A non-contrast CT scan of the elbow was performed preoperatively and in the subsequent postoperative follow-up. In all patients, there was a fracture of the olecranon, which required operative fixation using the posterior incision based on the Boyd approach. In all the cases, a fracture of the head of the radius was present. Fixation of the radial head fracture with a plate or headless screws was performed as the most appropriate treatment for each patient.

**Figure 1:**
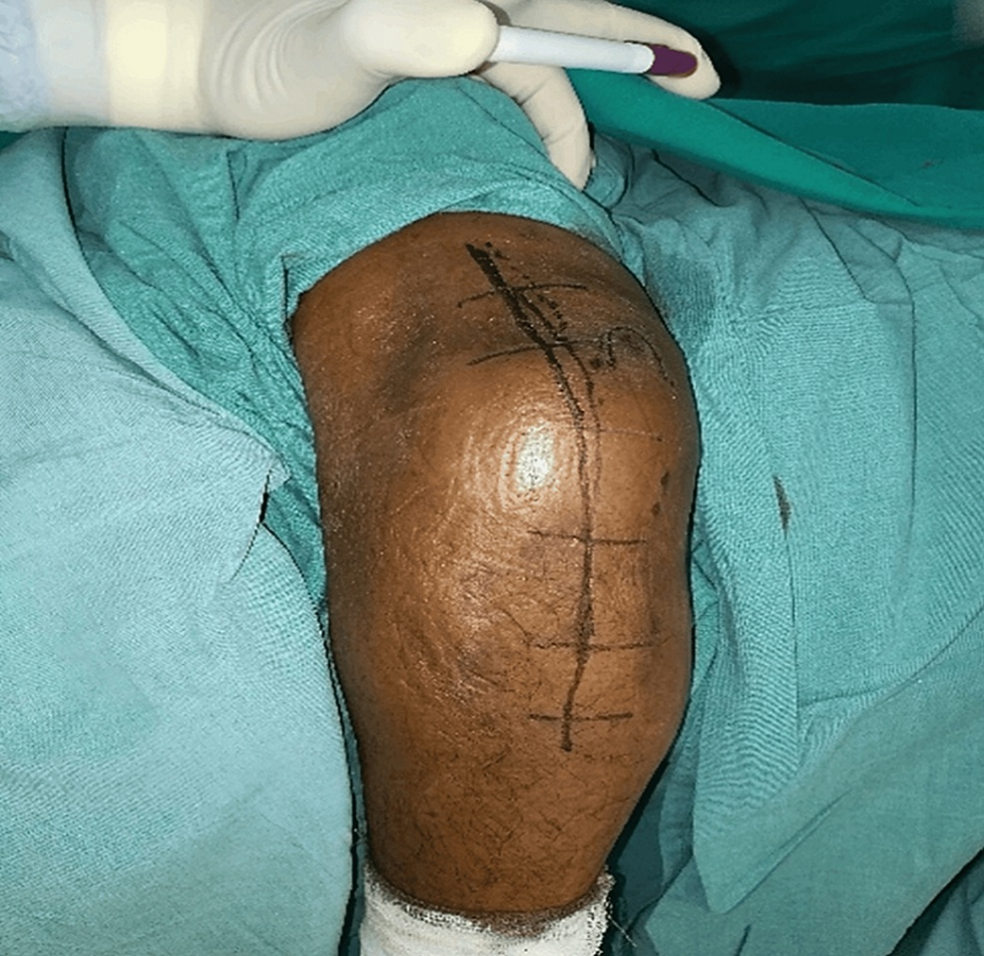
Line of incision

The approach was performed with the patient in the lateral position with the affected side up and rested over a L-bar. The skin incision was performed as per previous studies, beginning proximal to the elbow and lateral along the triceps tendon, followed by curving distally over the lateral aspect of the olecranon tip and continuing along the subcutaneous border of the ulna (Figure 1).

**Figure 2:**
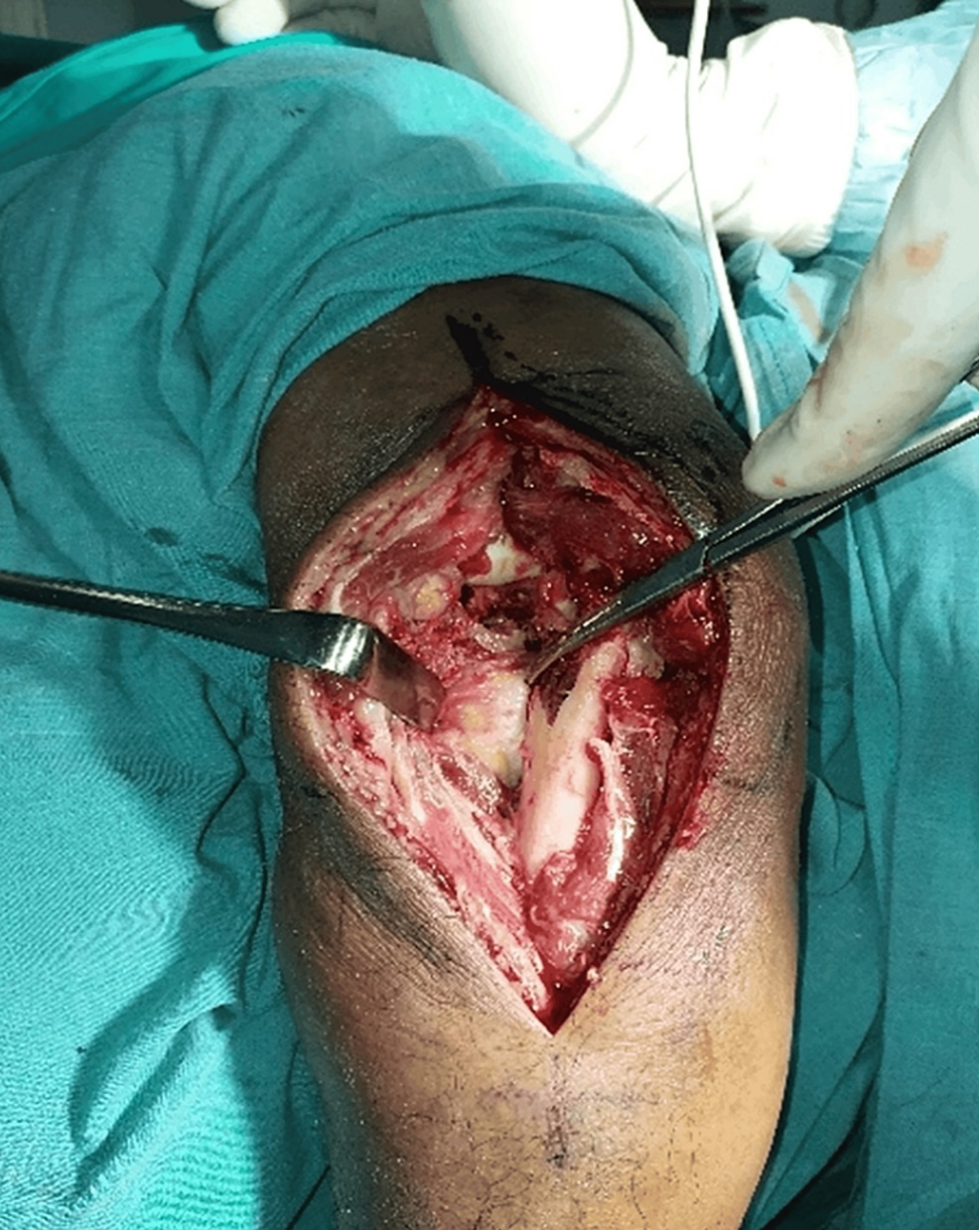
The deep fascia was incised in line with the incision to approach the lateral margin of the ulna between the anconeus insertion and the flexor carpi ulnaris

The deep fascia was incised in line with the skin incision to approximately the lateral border of the ulna between the attachment of the anconeus and the flexor carpi ulnaris muscle (Figure 2).

**Figure 3:**
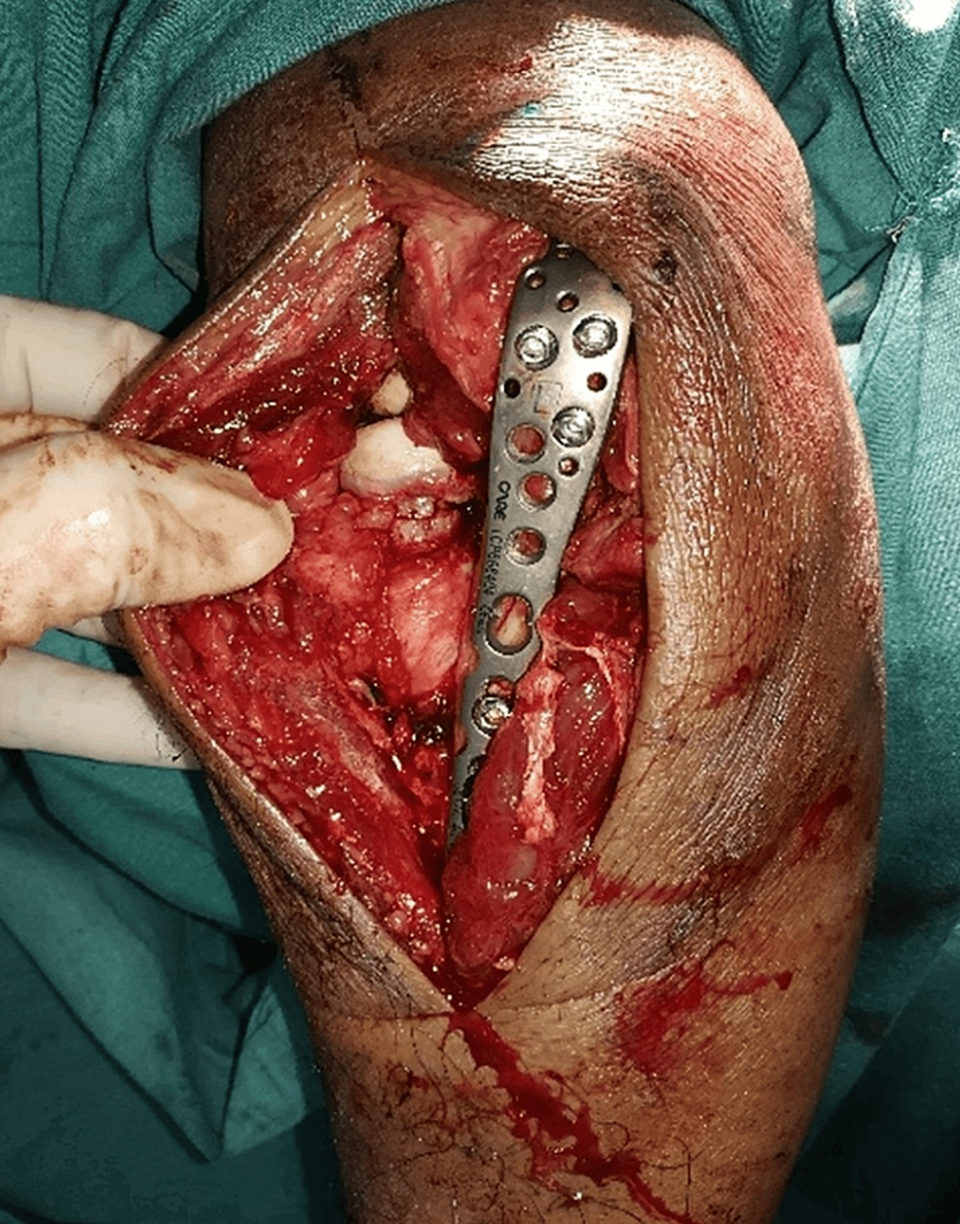
Posterior joint capsule over the radial head exposed and capsule and annular ligament reflected as single tissue mass

The anconeus muscle, followed by the supinator muscle, was separated from its ulnar attachment. The anconeus and supinator muscles were also released from the posterior part of the interosseous membrane, taking care to protect the PIN, which is present within the substance of the supinator muscle. In the next step, the retraction of these muscles exposed the posterior joint capsule near the head of the radius. The lateral ulnar collateral ligament (LUCL) was left undisturbed and attached to the capsule, and the annular ligament was then reflected as a single layer and primarily repaired later (Figure 3).

**Figure 4:**
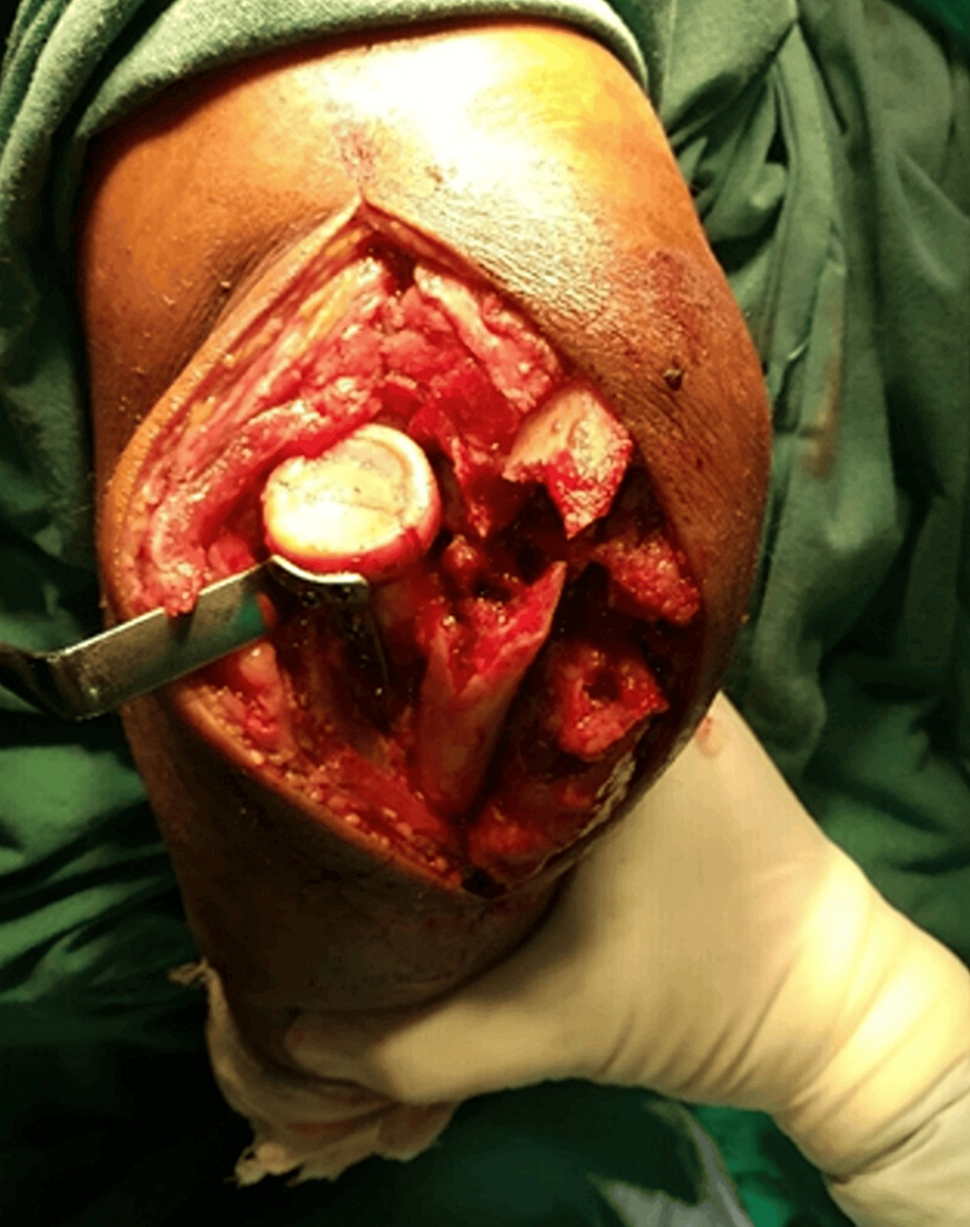
Radial head fixation done with T-plate

Olecranon fractures were fixed with locking plates for comminuted fractures and with dynamic compression plates for simpler fractures. Fixation of the radial head was performed with either headless screws or radial head plates, depending on the type of fracture (Figure 4).

After fixation of the fracture under the guidance of an image intensifier, the annular ligament was repaired. The elbow range of motion was checked intraoperatively. After the wound was closed, a plate was applied above the elbow for three days. The range of motion was initiated after three days.

Postoperative follow-up was performed at one, three, six, and 12 months. Patients were clinically assessed for elbow range of motion, level of pain, and stability of the elbow during each follow-up. After 12 months, each patient was also assessed with the Mayo Elbow Performance Index (MEPI). Serial radiographs were taken to assess fracture healing and post-traumatic osteoarthritis [1-5].

## Results

There were 16 patients in our study, and regarding the gender distribution, 12 patients were men and four were women. With respect to age, the mean age was 38.6 years, with an age range of 21 to 70 years. None of the patients had any wound healing problems or postoperative infections. All fractures healed well in satisfactory alignment, which was confirmed by radiographs during the serial follow-up. Two patients had mild elbow pain on range of motion during their final follow-up. There was no incidence of elbow instability. The flexion-extension movement was 135° ± 10° in the affected elbow compared to 145° ± 5° in the unaffected elbow. With regards to pronation-supination movement, it was 168° ± 12° in the affected elbow and 176° ± 3° in the unaffected elbow. Postoperatively, there was no significant difference in the range of motion values between the affected and unaffected elbows. The mean MEPI score was found to be 95 ± 5 (range 90 to 100). All our patients were satisfied and were able to return to their full activity levels. There was no incidence of post-traumatic arthritis of the elbow joint and synostosis of the radius and ulna. One of our patients had neuropraxia of the PIN, which fully recovered as evidenced during follow-up. The results of our study are illustrated in the table showing patient demographics, mode of injury, range of motion, and MEPI scores at follow-up (Table 1).

**Table 1:** Patient demographics, mode of injury, range of motion, and MEPI score at follow-up MEPI: Mayo Elbow Performance Index, RTA: road traffic accident

Patient	Age	Gender	Mechanism	Follow up months	Range of motion (flexion/extension in degree)	Range of motion (supination/pronation in degree)	MEPI score
1	24	M	RTA	14	140	160	95
2	70	M	RTA	14	135	170	95
3	42	F	Fall	16	130	165	90
4	22	M	RTA	12	140	180	95
5	28	M	Fall	14	135	170	100
6	55	M	Fall	12	125	180	95
7	38	M	RTA	16	145	165	100
8	29	F	RTA	14	140	170	95
9	21	M	RTA	14	135	170	95
10	49	M	RTA	12	140	180	90
11	32	M	Fall	16	130	165	90
12	29	F	RTA	12	140	160	95
13	62	M	Fall	14	130	170	95
14	23	M	RTA	16	135	170	100
15	27	F	RTA	12	130	160	90
16	68	M	Fall	14	140	165	100

## Discussion

There are a couple of approaches described for the exposure of the elbow for fractures of the radial head, terrible triad injuries, and other complex injuries of the elbow. The posterolateral approach, or Kocher approach, uses the interval between anconeus and extensor carpi ulnaris (ECU), and the lateral approach, or Kaplan's approach, uses the interval between extensor carpi radialis brevis and extensor digitorum communis. The lateral approach has the chance of damaging lateral structures of the elbow like the LUCL, common extensor origin, and PIN. The lateral and postero-lateral approaches are suitable for radial head fractures, but the main problem arises when there is a fracture of the olecranon or coronoid process along with a radial head fracture, particularly in terrible triad injuries and in cases of medial ligamentous injuries. In these cases, multiple incisions might be required if the lateral/postero-lateral approach is used, and this may lead to more recovery time post-surgery with an increased risk of elbow stiffness. The Boyd approach is excellent in these situations, particularly in cases where there is a need for exposure of the proximal radius, proximal ulna, and distal humerus, along with exposure of the ligamentous structures around the elbow. The MEPI score was used by most studies to check the outcome of elbow function postoperatively, and the MEPI score with the Boyd approach is quite good [2-14].

Complex elbow fractures generally occur in a wide range of populations, with high-energy trauma in young patients and low-energy falls in elderly patients. Many times, patients have multiple injuries that also include spinal injuries. So, repositioning may not be feasible for these patients. Also, good visualization of fracture fragments and less operative time are important for the fixation of these complex injuries. The Boyd approach maintains a single patient position. This single approach results in adequate exposure of the whole fracture, including the comminuted olecranon and radial head, with less operative time. The incision of this approach can be extended distally over the ulnar border to visualize the comminuted fracture extending to the proximal ulna. Radial head and neck fracture fixation with the plate can be done easily after elevating the supinator muscle from the ulna. The PIN traveling between the deep and superficial parts of the supinator muscle is protected after dissecting this muscle directly from the bone and keeping the forearm pronated. Diliberti et al. located the PIN safe zone to be 52 ± 7.8 mm along the lateral border of the proximal radius [1-2]. Our findings were consistent with them, and with proper dissection and technique, PIN can be protected from any injury.

Bauer et al. found the increased rates of synostosis of the radius and ulna fixed via the Boyd approach. The majority of these cases in their study, however, involved plate osteosynthesis of both the proximal radius and ulna. In our study, the Boyd approach was utilized for the fixation of an olecranon fracture with a radial head or neck fracture. We did not include the patients with head injuries, as this may increase the risk of synostosis. With the help of the Boyd approach, we were able to obtain proper fracture fixation, stability of the elbow joint, and annular ligament repair, thus allowing us to start the range of motion within three to five days. This early active motion prevents synostosis development and helps in the eventual return of functional range of motion. On follow-up, we had no cases of synostosis [1-3].

The study by Ayala et al. found heterotopic ossification in 5% of cases leading to restriction of range of motion, postoperative elbow instability in 2% of cases, and neuropathy in 11% of cases, mostly of the ulnar nerve [4]. Another study by Das et al. found elbow stiffness and neuropraxia of the ulnar and PIN as complications [5]. In our study, although we did not have any such complications, they cannot be ruled out as no follow-up beyond one year was done.

Robinson et al. used a modified Boyd approach by using the fascia between the anconeus and ECU, followed by supinator osteotomy and release of the annular ligament, on 21 patients with complex elbow fractures and had good functional outcomes. They also suggested that the Boyd approach gives good exposure to the lateral structures of the elbow and has a minimal chance of causing damage to the PIN [15].

## Conclusions

Many approaches have been described in the orthopedic literature for complex elbow fractures. For complex elbow fractures, there is no particular single incision approach through which we can get access to all three joints of the elbow (radiocapitellar, ulnohumeral, and superior radioulnar joint) except for the Boyd approach. This approach has various advantages, such as no repositioning of patients as required in the multiple incision approach, single-incision, less operative time, wide exposure useful for multiple fracture fragments, and minimal risk of injury to the neurovascular structures of the elbow apart from the posterior interosseus nerve, which can also be protected from injury intraoperatively by elevating the supinator muscle from the ulnar border while keeping the forearm pronated. However, there are some limitations to this study, like the short duration of follow-up, although osteoarthritis is a long-term complication, and the smaller number of patients in the study. More studies are needed in the future with a larger number of participants and a longer duration of follow-up to know about the outcomes.
